# Application of one-, three-, and seven-day forecasts during early onset on the COVID-19 epidemic dataset using moving average, autoregressive, autoregressive moving average, autoregressive integrated moving average, and naïve forecasting methods

**DOI:** 10.1016/j.dib.2021.106759

**Published:** 2021-01-15

**Authors:** Christopher J. Lynch, Ross Gore

**Affiliations:** Old Dominion University – Virginia Modeling, Analysis, and Simulation Center (VMASC), United States

**Keywords:** Coronavirus COVID-19, Infectious diseases, Epidemic modeling, ARIMA(p,d,q) model, ARMA model, Holt-winters exponential smoothing model, Statistical analysis, Short-range time series forecasting

## Abstract

The coronavirus disease 2019 (COVID-19) spread rapidly across the world since its appearance in December 2019. This data set creates one-, three-, and seven-day forecasts of the COVID-19 pandemic's cumulative case counts at the county, health district, and state geographic levels for the state of Virginia. Forecasts are created over the first 46 days of reported COVID-19 cases using the cumulative case count data provided by *The New York Times* as of April 22, 2020. From this historical data, one-, three-, seven, and all-days prior to the forecast start date are used to generate the forecasts. Forecasts are created using: (1) a Naïve approach; (2) Holt-Winters exponential smoothing (HW); (3) growth rate (Growth); (4) moving average (MA); (5) autoregressive (AR); (6) autoregressive moving average (ARMA); and (7) autoregressive integrated moving average (ARIMA). Median Absolute Error (MdAE) and Median Absolute Percentage Error (MdAPE) metrics are created with each forecast to evaluate the forecast with respect to existing historical data. These error metrics are aggregated to provide a means for assessing which combination of forecast method, forecast length, and lookback length are best fits, based on lowest aggregated error at each geographic level.

The data set is comprised of an R-Project file, four R source code files, all 1,329,404 generated short-range forecasts, MdAE and MdAPE error metric data for each forecast, copies of the input files, and the generated comparison tables. All code and data files are provided to provide transparency and facilitate replicability and reproducibility. This package opens directly in RStudio through the R Project file. The R Project file removes the need to set path locations for the folders contained within the data set to simplify setup requirements. This data set provides two avenues for reproducing results: 1) Use the provided code to generate the forecasts from scratch and then run the analyses; or 2) Load the saved forecast data and run the analyses on the stored data. Code annotations provide the instructions needed to accomplish both routes.

This data can be used to generate the same set of forecasts and error metrics for any US state by altering the state parameter within the source code. Users can also generate health district forecasts for any other state, by providing a file which maps each county within a state to its respective health-district. The source code can be connected to the most up-to-date version of *The New York Times* COVID-19 dataset allows for the generation of forecasts up to the most recently reported data to facilitate near real-time forecasting.

## Specifications Table

**Table:** 

Subject	Infectious Diseases
Specific subject area	Short-range forecasting methods applied to evaluate forecasting characteristics during early onset of COVID-19 case spread
Type of data	RDS (R data object) Graph (pdf, jpeg, eps) Rproject (software)
How data were acquired	The input data was acquired through “*us-counties.csv”* obtained from https://github.com/nytimes/covid-19-data on April 23, 2020 [1]. This provides cumulative COVID-19 case data up to April 22, 2020. Forecasts were created using Naïve, Growth, HW, MA(1), AR(1), ARMA(1,1), and ARIMA(*p,d,q*) forecasting methods. Values for *p* and *q* range from 1 to 3 and *d* range from 1 to 2. RStudio version 1.2.5033 and R version 3.6.3 were used to create the code and conduct analyses. Additionally, the R-packages *stats*[2], *base r package*[2], *forecast*[3], *ggplot2*[4], and *tidyverse*[5] are required to run the source code.
Data format	Data are raw, filtered, and analyzed. R project file and R script files (code) for execution in RStudio.
Parameters for data collection	Virginia county, health district, and state level forecasts are created using *The New York Times*’ COVID-19 cumulative case count data using: naïve; Growth; HW; MA(1); AR(1); ARMA(1,1); ARIMA(p,d,q). Forecast lengths include one, three, and seven days forward. Lookback lengths of one, three, seven, and all prior days. Error metrics calculated using MdAE and MdAPE and are in units of cumulative COVID-19 cases.
Description of data collection	Using the cumulative COVID-19 case count data from The New York Times, we filter for “Virginia” and we provide a mapping of VA's counties to their health districts. For each of the 46 days containing non-zero case counts, up to 12 forecasts are generated for each day using combinations of one, three, and seven days forward and one, three, seven, and all days prior. This repeats for all 7 forecasting methods to produce 1329,404 forecasts. The MdAE and MdAPE of each forecast is calculated and aggregated by forecast type to evaluate each method's performance at forecasting cumulative case counts.
Data source location	Old Dominion University – Virginia Modeling, Analysis, and Simulation Center Suffolk, VA, U.S.A.
Data accessibility	The data is hosted in a public repository and entitled: “*Short-range Early Phase COVID-19 Forecasting R-Project and Data*” [6] and made available under the MIT license. Repository name: Mendeley Data Data identification number: 10.17632/cytrb8p42g.2 [6] Direct URL to data: https://data.mendeley.com/datasets/cytrb8p42g/2 Instructions for accessing these data: Access, download, and extract the data package from the provided URL. Using RStudio, open the RProject file "short-range-early-onset-covid-19-forecasting.Rproj". This automatically sets RStudio's working directory to the location of your extracted folder. All needed data files are included within this package and the file paths are relative to the location of the RProject location. No changes (e.g. path name updates) are required to get started. Within RStudio, open "forecasting_article_code.R". This file is the primary file for this data. This file contains annotations to direct the user through the code. This includes what is being forecast, how the forecasts are generated, and all of the analytical steps taken. Running each line of code in this file recreates the data set. The code provides all steps taken to generate the figures contained within the package. Copies of the files created by the code are included within the package and can directly be loaded into the code. This removes the requirement to run the forecasts, as this can be time-consuming and resource-intensive. This allows the user to move straight through to the analysis section of the code. Annotations within the code provide direction on how to properly navigate this process.
Related research article	C.J. Lynch, R. Gore, Short-range forecasting of coronavirus disease 2019 (COVID-19) during early onset at county, health district, and state geographic levels: Comparative forecasting approach using seven forecasting methods, J. Med. Internet Res. In Press. [7]

## Value of the Data

• These data are useful as they provide short-term forecasts of cumulative COVID-19 case counts during early onset to extend the knowledge base of COVID-19 disease spread at three different levels of granularity: state, health-district and county. MdAE and MdAPE error metrics are utilized to evaluate the forecasts with respect to historical data to inform validation and forecasting method selection.
• Researchers, institutions, and health officials involved in preventing COVID-19 spread can benefit from these data by identifying which method produces the smallest error for an area and utilizing that method to generate future short-term forecasts for that area. This data is reusable and the source code can be extended to different states, health districts, and counties or any other level of geographic granularity.
• These data can be reused to: (1) generate forecasts within other states by changing or removing the “Virginia” filter; (2) generate forecasts of current dates by accessing the up-to-date data from *The New York Times*; and (3) applying these forecasting methods and error metrics to other geographic levels and/or locations by providing the relevant time series formatted data on case counts.
• These data can aid policy makers and researchers in addressing questions of selecting optimal *p, d*, and *q* parameters for AR, MA, ARMA, and ARIMA forecasting techniques by providing benchmarks for how well each of these techniques perform for a geographic location.
• Members of the public can utilize these short-range forecasts to gain insight into their expected local-area COVID-19 case counts over the upcoming week. This provides an additional source of information to inform individuals in making decisions or creating plans for engaging in activities or interacting within their communities over the next few days.
• Policy makers of regions that do not fit solely on the county or state levels can generate forecasts based on their areas of influence to obtain a direct representation of expected case counts combined with statistically significant support on which forecasting methods are producing the smallest error within that area.

## 1 Data Description

### 1.1 Coronavirus Mendeley data package folder

This root package includes all of the data pertaining to this project. The top level of this package includes a licensing information document (matching the license included in this article), a *README* file provides a description of the dataset, and a folder containing all of the code, data, and figures entitled *Short-range Early Phase COVID-19 Forecasting R-Project*.

### 1.2 short-range-early-onset-covid-19-forecasting Rproj file

This is the starting point for this dataset. Within RStudio, select “File -> Open Project in New Session…” to open this Rproj file. This opens the project and sets RStudio's working directory to the current location of the Rproj file. All coded file paths are relative to the folder containing the RProject file; this facilitates replication by enabling the code to be executed without modification. The following files are presented in the order in which they are utilized within the project.

### 1.3 forecasting_article_code R file

This serves as the baseline file for this dataset. This file is heavily annotated with comments in the source code to instruct users on the process for generating forecasts, loading data from the *data* folder, and performing the analyses. All of the calls to generate the Naïve, Growth, HW, MA(1), AR(1), ARMA(1,1), and ARIMA(p,d,q) forecasts occur within this file; however, the functions for generating these forecasts appear within the following files.

### 1.4 import_county_hd_state_cum_case_counts R file

This code loads the stored copy of The New York Times cumulative case count data [5] as of April 23, 2020. The data is then filtered for Virginia. A mapping of the VA county names to their corresponding health districts is also loaded and formatted into time series data objects.

### 1.5 forecasting_article_functions R file

This file contains a majority of the functions needed to generate the county level forecasts. County level time series objects are generated using the imported case count data. The Naïve, HW, MA(1), AR(1), ARMA(1,1), and ARIMA(p,d,q) forecasts are generated within this file. ARIMA's values for *p* range from 1 to 3, *d* ranges from 1 to 2, and *q* ranges from 1 to 3. Combinations of one-, three-, and seven-day forecast lengths and one-, three-, seven-, and all-prior days cumulative case information for the county, health district, and state levels. The code for producing corresponding *P*-values reflecting statistically significant outcomes is also contained within this file.

Alternatively, the provided forecasts can be loaded directly from the *data* folder. Annotations within the code provide instruction on how to properly run the forecasts or load the existing forecasts. This option is provided as the generation of the forecasts is time consuming and resource intensive.

### 1.6 forecasting_article_functions_state_and_district R file

This file contains the functions needed to generate the health district and state level forecasts. State level time series objects are generated using the imported case count data. The Naïve, HW, MA(1), AR(1), ARMA(1,1), and ARIMA(p,d,q) forecasts are generated for health districts and the state. ARIMA's values for *p* range from 1 to 3, *d* ranges from 1 to 2, and *q* ranges from 1 to 3. Due to high time and resource requirements, the provided forecasts can be loaded directly at this stage.

### 1.7 function-version-extrapolate-seven-days-for-each-forecast R file

Generates the Growth forecasts at the county, health district, and state levels. It determines the current rate of growth for the area based on the number of new cases in the last 24 h within the respective geographic area and number of new cases in the state. Then a forecast is generate using this rate of growth for the geographic area and desired forecast length (one, three, and seven days).

### 1.8 compute-infections-per-county R file

Provides functions necessary helper functions for the creation of the Growth forecasts. This file is sourced by function-version-extrapolate-seven-days-for-each-forecast.

### 1.9 Data folder

This folder contains three additional folders of data: (1) *generated-table-data*; (2) *generated-time-series-data*; and (3) *input-data*. The files contained in these folder allow for reproducibility and transparency in the generated data.

1. generated-table-data contains 31 data files that include the MdAE tables, the MdAPE tables, the aggregated validation tables, and a readme file. Each table exists at a single geographic level (county, health district, or state) as indicated in its title. These are the filtered data files that facilitate analysis. These files can be imported directly in the code to prevent having to run the code to directly generate these tables, as this can result in lengthy run times and have high resource demands. The MdAE and MdAPE metrics calculated from the aggregated combinations of forecasting method, lookback length, and forecast length include: the median scores of the upper and lower notch values; the median of the aggregated median values; the median scores of the upper and lower whiskers; the median scores of the upper and lower quartile ranges; and the number of data observations.
2. generated-time-series-data contains 75 data files providing the raw 1329,404 generated forecasts. These are separated by geographic level and forecasting method as indicated in their titles.
3. input-data contains two data files: (1) us-counties.csv; and (2) Health-District-County-Info.RDS. A snapshot of The New York Times COVID-19 dataset [5] on April 23, 2020 is provided in us-counties.csv. This provides county-level cumulative case count information through April 22, 2020. This file is provided to ensure reproducibility of the created data. The Health-District-County-Info.RDS file provides a mapping of VA's county names to their respective health districts to enable aggregation of county data to the health district level to allow for forecasting.

The seven forecasting methods vary in their underlying assumptions about their relationships to the means or trends in prior observations and forecasted dates. Table 1 provides descriptions of each forecasting method, the primary assumptions associated with each method, and identifies the R packages that were utilized in their code implementation.

**Table 1:** Description and primary assumptions of each of the forecasting methods utilized along with the R-packages utilized to for implementation.

	Description	Primary Assumptions	R Packages Utilized
Naïve	The forecasted value for each day being forecasted is equal to the current day's value. This forecast method makes no use of prior knowledge to inform the future values and serves as a benchmark for comparisons.	Since the data pertains to cumulative case counts, this forecast assumes no change for each forecasted day from the present value [8].	*stats*[2]
Holt-Winters exponential smoothing (HW)	Holt-Winters exponential smoothing assumes exponentially decreasing weights over the prior *k* observations.	Assumes higher weighting to the recent past with exponentially decreasing weighting given to each successive prior *k* observations [9,10].	*stats*[2]
Growth Rate (Growth)	The forecast growth rate is calculated as follows: (1) initial per county growth rate is based on the increased number of cases to the prior day of the forecast start date; (2) state level growth rate is the cumulative increase in cases since the prior day for the entire state; (3) *n* forecasts are generated by uniformly sampling *n* times between the initial county growth rate and the state level growth rate; and (4) the final forecast is the average of the *n* forecasts [11].	Assumes a linear relationship between the forecast dates and the prior date's count. All weighting is given to the date prior to the forecast's start date.	*base-package*[2]
Moving Average (MA)	For univariate time series, simple MA models depend linearly on the current value and past *k* observations [8].	Assumes a stationary mean along with equal weighting for all prior *k* observations.	*forecast*[3]
Autoregressive (AR)	AR models assume a linear dependency between the forecasted values and the current and prior *k* observations along with a stochastic component to account for behavior.	Assumes that forecasted values are based on a linear combination of prior values [8]. Does not assume observations are stationary due to stochasticity.	*stats*[2]
Autoregressive Moving Average (ARMA)	ARMA models are a combination of MA and AR components. The AR component involves regressing the variables based on their own lagged values [12]. MA models the error as a linear combination of error terms at various points in the past.	Assumes observations are at least weakly stationary.	*stats*[2]
Autoregressive Integrated Moving Average (ARIMA)	ARIMA models apply to non-stationary data and consist of a combination of MA and AR components along with a difference measure to make the data stationary [8].	Assumes observations are non-stationary.	*stats*[2]

### 1.10 Figures folder

This folder contains the figures generated throughout the execution of the *forecasting_article_code* file. Figures are generated in three non-interactive formats (vector and non-vector graphics) as well as an interactive HTML format for a total of 28 image files. These files provide box plot comparisons of the MdAE information for the seven forecasting methods at the county (*County Forecasts MdAE*), health district (*Health District Forecasts MdAE*), and state (*State Forecasts MdAE*) levels that separate the information based on forecast length (one, three, and seven days) and lookback length (one, three, seven, and all prior days). Box plot comparisons of the MdAPE data are provided in the *Aggregate Forecasts MdAPE* figure. This figure groups data by forecasting method and plot the aggregated values at each geographic level for a total of 21 box plots divided into 7 groups. The same process is utilized to generate figures for comparing the ARIMA(p,d,q) outcomes and use a naming convention of *County Level ARIMA(p,d,q) Forecasts MdAE*.

## 2 Experimental Design, Materials and Methods

**Fig. 1:**
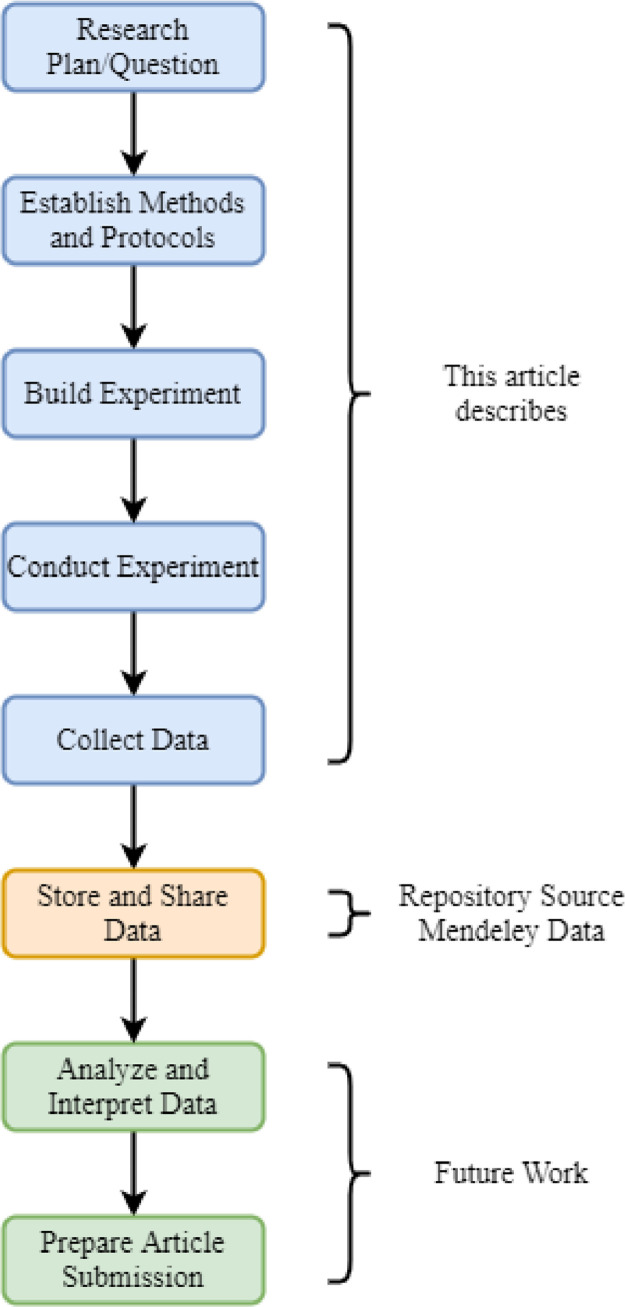
High level overview describing how this article fits within the context of the experimental design, historical data, the data repository storage location, and future work.

We create one-, three-, and seven-day forecasts of cumulative COVID-19 case counts at the county, health district, and state levels using Naïve, Growth, HW, MA(1), AR(1), ARMA(1,1), and ARIMA(p,d,q) forecasting methods. Then, we generate MdAE and MdAPE error metrics to utilize for validating the forecasts against historical data. This section describes the experimental design, the forecasting methods, the obtained county-level historical data, validation criteria, and the validation data generated. For a description of the data, refer to the previous section. The presentation and discussion of findings based on this data is outside the scope of this article. Fig. 1 distinguishes the role of this article from the roles of the data stored in the repository and future publications.

**Fig. 2:**
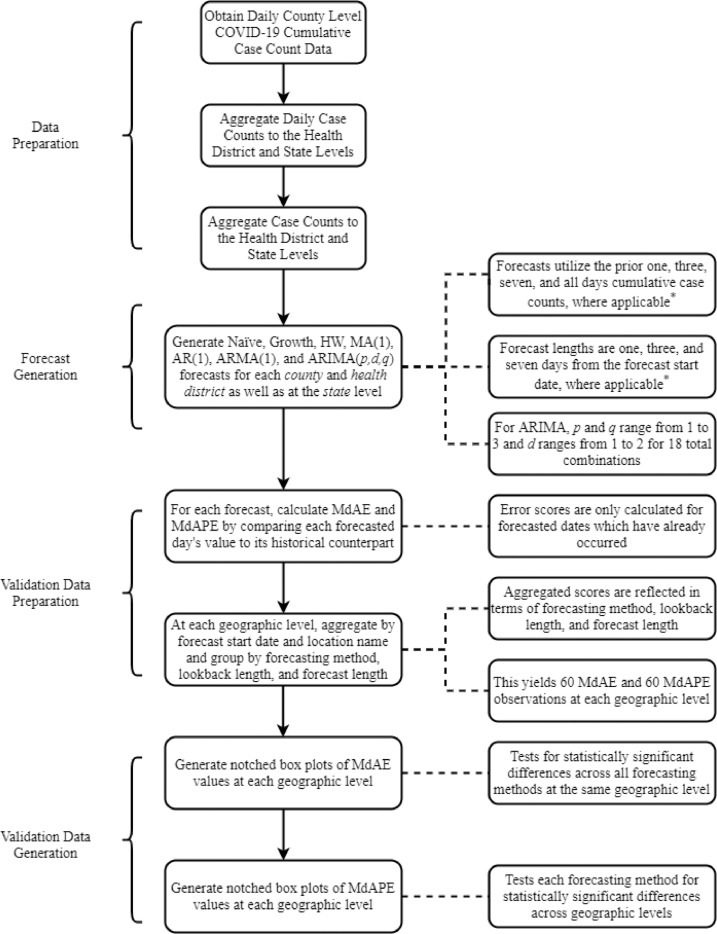
Experimental design for preparing the historical data, generating forecasts, and preparing and generating data to use for validation. *Where applicable represents that forecasts are only generated when the dates covered by both the lookback and forecast lengths exist.

The experimental design falls into four primary categories: data preparation; forecast generation; validation data preparation; and validation data generation. Data preparation describes how the historical data is obtained and how that data is aggregated to the health district and state geographic levels. Forecast generation describes the assumptions pertaining to each of the selected forecasting methods. Validation data preparation describes the error metrics selected to measure how well each forecast performs compared to the historical data and how the forecasted COVID-19 cumulative case counts are aggregated to facilitate validation. Validation data generation describes the validation methodology and the process for conducting comparisons. Fig. 2 provides a breakdown of the experimenal design.

In the first stage, we conduct data preparation using *The New York Times*’ COVID-19 cumulative case count data [1]. This county-level data is filtered to only include the 133 counties and independent cities within the state of VA. Each county's cumulative case counts per day are then stored as time series objects. Using the assigned health district mappings per county from the VA Department of Health [13], we aggregate each county's cumulative case counts within each of the 35 health districts and store them as time series objects. Finally, we aggregate the cumulative case counts from every county each day to form a time series object for the entire state of VA.

In the second stage, we generate forecasts using combinations of the seven forecasting methods, four lookback lengths, and three forecast lengths. Lookback lengths use one-, three-, seven-, and all-prior days information up to the date of first reported case per geographic location. Forecast lengths are one, three, and seven days ahead. To fulfill the validation criteria in the following step, forecasts are only generated over periods where historical data exists. As such, for this dataset, April 15th, 19th, and 21st, 2020, are the final dates for generating seven-, three-, and one-day forecasts, respectively. To generate forecasts for each county, health district, and the state, we utilize the time series objects from the first stage.

1. For each combination of lookback length (x) and forecast length (y), every date within each time series is checked to determine if the necessary historical data exists in both directions.
2. If yes, then a forecast of length y is generated and stored.
3. If no, then the forecast is skipped.

The process used to generate forecasts using each forecasting method follows. The utilization of the *k* prior days and *j* forecasted days ahead are indicated.

• Naïve

For each county, the prior single day's value is the forecast value for each of the next j days.

For each health district, the aggregated sum of each of its counties’ prior single day's cumulative case counts become the forecast value for each of the next j days.

For VA, the aggregated sum of all cumulative case counts from the prior single day becomes the forecast value for each of the next j days.

• HW

For each county, Holt-Winters exponential smoothing of the prior k day's values are used to forecast the values for the next j days.

For each health district, the aggregated sum of its counties’ cumulative case counts for each of the prior k days are used to forecast values for the next j days.

For VA, the aggregated sum of all cumulative case counts within VA's counties over the prior k days are used to forecast the next j days.

• Growth

For each county, the prior one day's value is used to calculate the current growth rate for the county over the following j days. Then, the prior day's values for all the counties are used to calculate the growth rate for VA for the same j days. A group of n forecasts are generated for the county by uniformly sampling a growth rate between the county's and VA's rates. The average of the n forecasts is utilized as the final forecast for the county.

Each health districts’ forecasts are generated using this same method for each of its counties and then summing the values for each of the j forecasted days.

VA's forecasts are generated by forecasting each county's values using this same method and then summing all the counties’ values for the state for each of the j forecasted days.

• MA, AR, ARMA, and ARIMA

For each county, an equal weighting of the prior k day's cumulative case counts is used to forecast the next j days.

For each health district, the aggregated sum of its counties’ cumulative cases for each of the prior *k* days are used to forecast the next *j* days.

For VA, the aggregated sum of all cases within VA's counties for the prior *k* days are used to forecast the next *j* days.

We utilize a first order MA(1) model for all MA forecasts, a first order AR(1) model for all AR forecasts, first order AR and MA components for the ARMA(1,1) model for all ARMA forecasts, and an ARIMA(p,d,q) model. ARIMA's values for *p* range from 1 to 3, *d* ranges from 1 to 2, and *q* ranges from 1 to 3 for a total of 18 ARIMA forecasting models.

In the third stage, we prepare for validation by generating the error metrics needed to validate the forecasts against the historical data. To this end, we utilized MdAE and MdAPE as established metrics for evaluating forecasting error [14], [15], [16], [17]. Median-based metrics are applied as the forecasts contain outliers [14,15]. MdAE is scale dependent [16] and well suited for making comparisons between forecasts of the same scale [17]. MdAPE is applied to compare forecasts of differing scales [16]. We utilize MdAE to test for significant differences between forecasts at the same geographic level based on their performance with respect to the recorded historical values. We utilize MdAPE to measure the percentage difference of the forecasts from their observed historical values across geographic levels to account for different magnitudes of cumulative case counts when aggregating county-level data to health district and state-level geographic representations.

The process taken to prepare the validation data at each geographic level follows:

1. For each forecast, calculate baseline MdAE and MdAPE scores by comparing each forecast's set of values to their recorded historical counterparts
2. At each geographic level, aggregate the MdAE and MdAPE scores by forecast start dates and grouping results by location name, forecasting method, lookback length, and forecast length
  a. For each geographic level, store these results in a separate dataset
  b. This results in three tables for MdAE values and three tables for MdAPE values
3. Aggregate each of these six tables by location names and group by forecasting method, lookback length, and forecast length.
  a. This yields six datasets of aggregated MdAE and MdAPE values that convey the aggregated values based on each combination of forecasting method, lookback length, and forecast length independent of start date or location
  b. Sixty aggregated observations are created at each geographic level for a total of 180 observations for MdAE and 180 observations for MdAPE
  c. Note, this second level of aggregation is not applied to the state level data as only a single state, VA, is present in the dataset

In the fourth stage, we take the prepared validation data and we generate the aggregated error metrics needed to validate the generated forecasts. We utilize a method of notched box plots to identify statistically significant differences between the forecasting methods [18,19]. Notched box plots display the median values of the data along with notches representing the 95% confidence interval for each median represented as ±1.58*(Interquartile Range)/sqrt(n) for the upper and lower notches [20,21]. Comparisons of box plots resulting in non-overlapping notch ranges are considered to be statistically significantly different with an alpha of 0.05 [18]. We use the *geom_boxplot* package within *ggplot2* [4] to construct the notched box plot data for the aggregated forecast data. The median values for each of the MdAE and MdAPE metrics for each forecasting method, lookback length, and forecast length combination are produced as the output of this stage. For consistency checking, P-values are also generated during this phase using Mood's Median test to test for statistically significant differences in medians between forecasting combinations.

## Ethics Statement

The authors declare that this work does not involve the use of human subjects or experimentation with animals.

## CRediT Author Statement

**Christopher J. Lynch:** Conceptualization, Methodology, Software, Validation, Data Curation, Writing – Original Draft, Visualization, Project administration; **Ross Gore:** Conceptualization, Methodology, Software, Validation, Data Curation, Writing – Review & Editing, Supervision.

## Declaration of Competing Interest

The authors declare that they have no known competing financial interests or personal relationships which have, or could be perceived to have, influenced the work reported in this article.

## Data Availability

Short-range Early Phase COVID-19 Forecasting R-Project and Data (Original data) (Mendeley Data).

## References

[1] The New York Times, Coronavirus (Covid-19) Data in the United States, GitHub, https://github.com/nytimes/covid-19-data, 2020 (retrieved 23 April 2020).

[2] R Core Team. R: a language and environment for statistical computing. In: R Found. Stat. Comput. Vienne, Austria. https://www.R-project.org/, 2020 (accessed 30 March 2020).

[3] Hyndman R.J., Khandakar Y. (2008). Automatic Time Series Forecasting: the Forecast Package for R. J. Stat. Softw..

[4] Wickham H. (2016). ggplot2: Elegant Graphics For Data Analysis.

[5] Wickham H., Averick M., Bryan J., Chang W., McGowan L.D., François R., Grolemund G., Hayes A., Henry L., Hester J., Kuhn M. (2019). Welcome to the Tidyverse. J. Open Source Softw..

[6] C.J. Lynch, Gore, R., Short-range early phase COVID-19 forecasting R-project and data, Mendeley Data, V2, doi: 10.17632/cytrb8p42g.2, 2020, https://data.mendeley.com/datasets/cytrb8p42g/2.

[7] C.J. Lynch, R. Gore, Short-range forecasting of coronavirus disease 2019 (COVID-19) during early onset at county, health district, and state geographic levels: comparative forecasting approach using seven forecasting methods, J. Med. Internet Res. (In Press). 10.2196/24925 PMC7990039 33621186

[8] Hyndman R.J., Athanasopoulos G (2018). Forecasting: Principles and Practice.

[9] Holt C.C. (2004). Forecasting seasonals and trends by exponentially weighted moving averages. Int. J. Forecast..

[10] Winters P.R. (1960). Forecasting sales by exponentially weighted moving averages. Manag. Sci..

[11] Jacquez J.A., O'Neill P. (1991). Reproduction numbers and thresholds in stochastic epidemic models I. Homogeneous populations. Math. Biosci..

[12] Ives A.R., Abbott K.C., Ziebarth N.L. (2010). Analysis of ecological time series with ARMA (p, q) models. Ecology..

[13] Virginia Department of Health, Local health districts. https://www.vdh.virginia.gov/local-health-districts/, 2020 (accessed 30 March 2020).

[14] Armstrong J.S., Collopy F. (1992). Error measures for generalizing about forecasting methods: empirical comparisons. Int. J. Forecast..

[15] Armstrong J.S., Armstrong J.S. (2001). Evaluating forecasting methods. Principles of Forecasting: A Handbook for Researchers and Practitioners.

[16] Hyndman R.J., Koehler A.B. (2006). Another look at measures of forecast accuracy. Int. J. Forecast..

[17] Shcherbakov M.V., Brebels A., Shcherbakova N.L., Tyukov A.P., Janovsky T.A., Kamaev V.Ae. (2013). A survey of forecast error measures. World Appl. Sci. J..

[18] McGill R., Tukey J.W., Larsen W.A. (1978). Variations of box plots. Am. Stat..

[19] Krzywinski M., Altman N. (2014). Visualizing samples with box plots. Nat. Methods..

[20] R.L. Nuzzo. The box plots alternative for visualizing quantitative data. PM&R. 2016 Mar; 8(3):268–272. 10.1016/j.pmrj.2016.02.001. 10.1016/j.pmrj.2016.02.001 26892802

[21] Chambers J.M., Cleveland W.S., Kleiner B., Tukey P.A. (1983). Graphical Methods for Data Analysis.

